# Supplementary material for: Dual Targeting of EZH2 Degradation and EGFR/HER2 Inhibition for Enhanced Efficacy against Burkitt’s Lymphoma
Source: Cancers (Basel). 2023 Sep 8;15(18):4472. doi: 10.3390/cancers15184472 (PMC10526300; doi:10.3390/cancers15184472)
Supplement: Supplementary file 1 [file cancers-15-04472-s001.zip › cancers-2550457-supplementary.pdf]

Supplementary Figures

Suppplementary figure S1

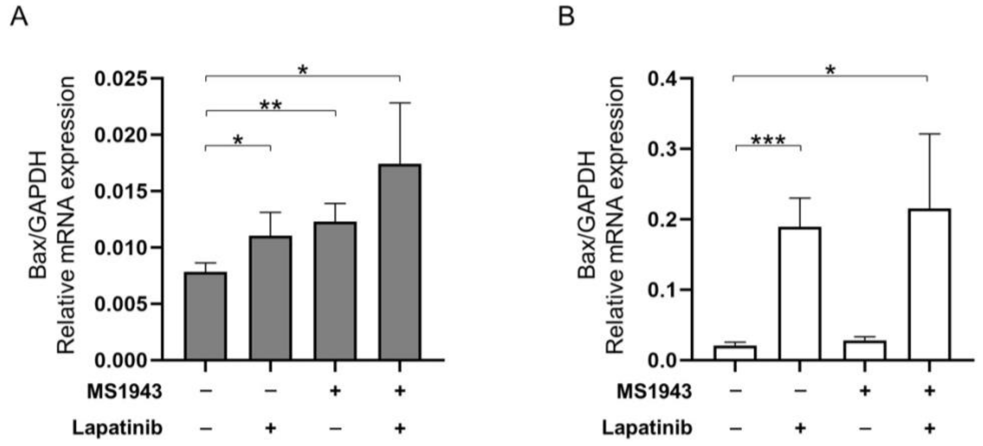

Figure S1. Combination of therapeutic agents induced upregulation of Bax by qRT-PCR. (A) Relative mRNA expression of Bax increased in combination treatment, indicating apoptosis in the Ramos cell line. (B) In Daudi cell line, relative mRNA expression of Bax is signicantly upregulated in Lapatinib and dual-treatment. This indicated dual-targeting approach has more efficacy leading apoptotic results.
*, $p < 0.05$; **, $p < 0.01$; ***, $p < 0.001$; as determined by two-tailed, unpaired t-tests. Error bars are shown as mean ± SD.

Suppplementary figure S2

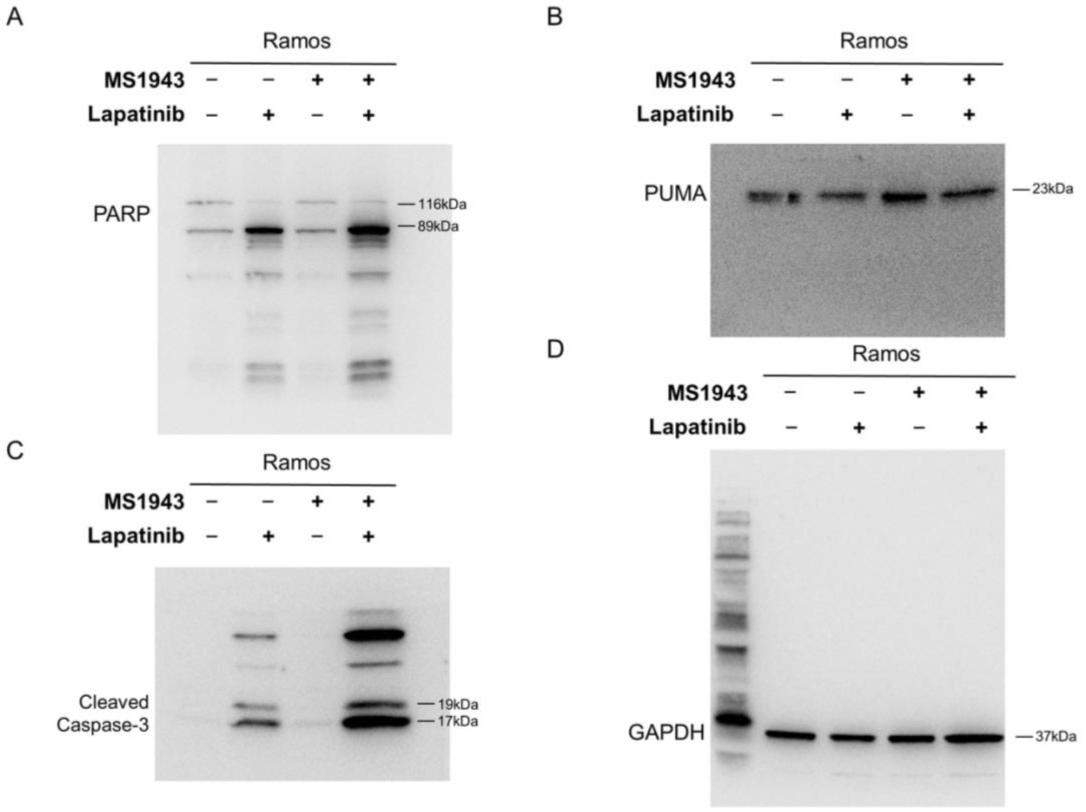

Figure S2. Western blot total gel results for apoptosis-related proteins in Ramos cell line. (A-C) Combination treatment induces upregulation of apoptotic proteins such as PARP, PUMA, and Cleaved Caspase-3. (D) Housekeeping protein GAPDH was detected by Western blotting.

Suppplementary figure S3

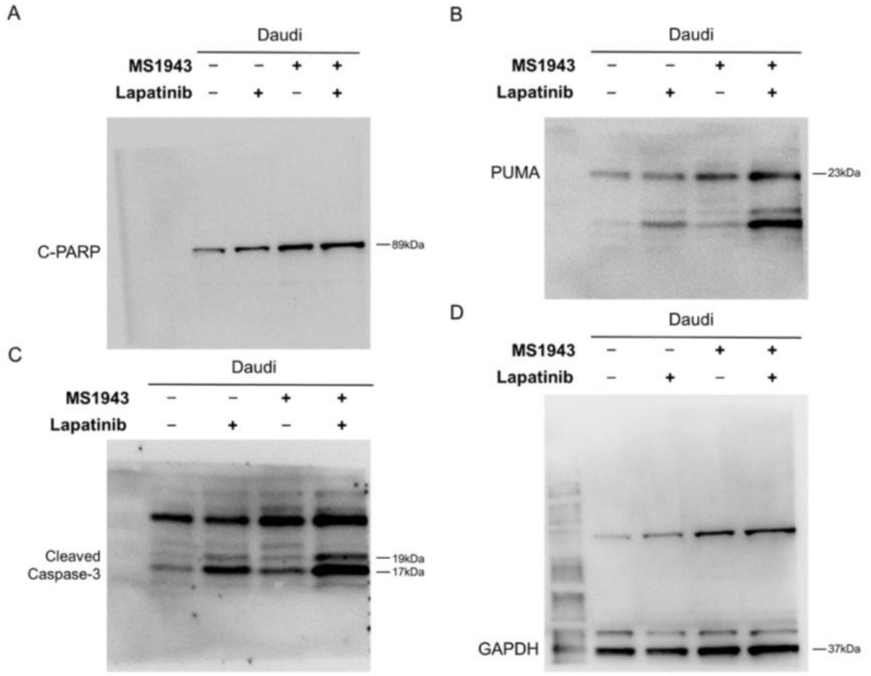

Figure S3. Western blot total gel results for apoptosis-related proteins in Daudi cell line. (A-C) Combination treatment induces upregulation of apoptotic proteins such as C-PARP, PUMA, and Cleaved Caspase-3. (D) Housekeeping protein GAPDH was detected by Western blotting.

Suppplementary figure S4

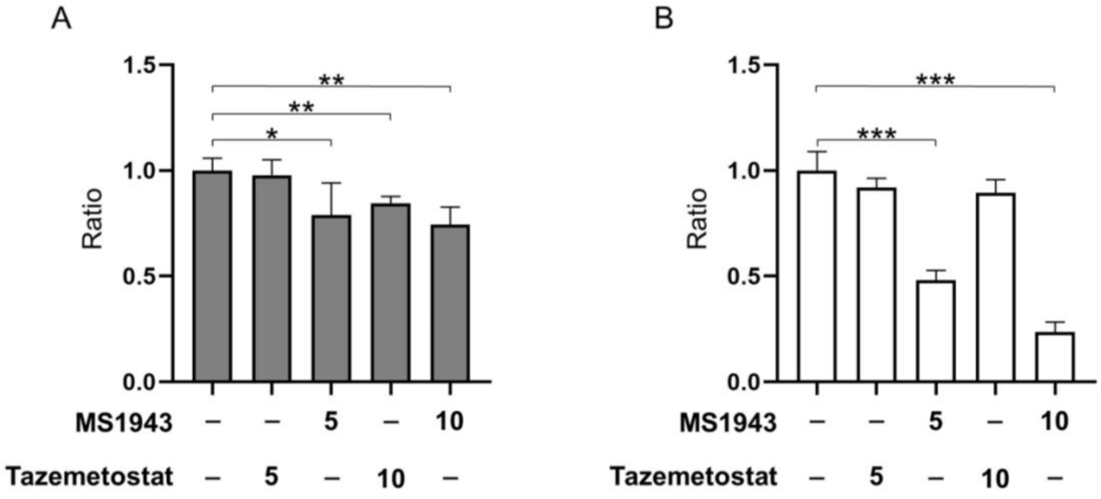

Figure S4. Cell viability after treatment with MS1943 and Tazemetostat was measured using

the CCK-8 assay. (A) The Ramos cell line was exposed without MS1943 and Tazemetostat (EZH2 inhibitor), and with 5 μM and 10 μM of MS1943 and Tazemetostat, respectively for 72 h. Data indicated MS1943 has more efficacy than Tazemetostat. (B) The Daudi cell line was exposed same as the Ramos cell line. This result showed MS1943 induced significant reduction compared to Tazemetostat. . *, $p < 0.05$; **, $p < 0.01$; ***, $p < 0.001$ as determined by two-tailed, unpaired t-tests. Error bars are shown as mean ± SD.
